# Metagenomic Predictions: From Microbiome to Complex Health and Environmental Phenotypes in Humans and Cattle

**DOI:** 10.1371/journal.pone.0073056

**Published:** 2013-09-04

**Authors:** Elizabeth M. Ross, Peter J. Moate, Leah C. Marett, Ben G. Cocks, Ben J. Hayes

**Affiliations:** 1 Biosciences Research Division, Department of Environment and Primary Industries, Bundoora, Victoria, Australia; 2 Dairy Futures Cooperative Research Centre, Bundoora, Victoria, Australia; 3 La Trobe University, Bundoora, Victoria, Australia; 4 Future Farming Systems Division, Department of Environment and Primary Industries, Ellinbank, Victoria, Australia; University of Illinois, United States of America

## Abstract

Mammals have a large cohort of endo- and ecto- symbiotic microorganisms (the microbiome) that potentially influence host phenotypes. There have been numerous exploratory studies of these symbiotic organisms in humans and other animals, often with the aim of relating the microbiome to a complex phenotype such as body mass index (BMI) or disease state. Here, we describe an efficient methodology for predicting complex traits from quantitative microbiome profiles. The method was demonstrated by predicting inflammatory bowel disease (IBD) status and BMI from human microbiome data, and enteric greenhouse gas production from dairy cattle rumen microbiome profiles. The method uses unassembled massively parallel sequencing (MPS) data to form metagenomic relationship matrices (analogous to genomic relationship matrices used in genomic predictions) to predict IBD, BMI and methane production phenotypes with useful accuracies (r = 0.423, 0.422 and 0.466 respectively). Our results show that microbiome profiles derived from MPS can be used to predict complex phenotypes of the host. Although the number of biological replicates used here limits the accuracy that can be achieved, preliminary results suggest this approach may surpass current prediction accuracies that are based on the host genome. This is especially likely for traits that are largely influenced by the gut microbiota, for example digestive tract disorders or metabolic functions such as enteric methane production in cattle.

## Introduction

The metagenome, the mix of DNA from all species in a sample, has recently become an area of great interest [1], [2], as the human body contains 10 times more bacterial cells than human cells [3]. This ratio is even more dramatic in cattle, with approximately 120 times more bacterial than bovine cells in each animal (File S1), reflecting the essential role of rumen microbial fermentation in converting low quality feed stuffs into meat and milk. A number of studies have explored the complexity of the human microbiome by sequencing metagenomes from all over the human body [4]–[9], and some from other species with examples including representatives of carnivores [10]–[12], omnivores [13]–[15] and herbivores [16]–[22]. Some of these sequences have been accompanied by host phenotypes (e.g. IBD [8] or BMI [7], [8], [23], [24]). These studies, and subsequent reanalyses of the data [25], [26], have reported species abundances and cluster analyses that associate the microbiome with the trait of interest. Given the complexity of the microbial communities, particularly in the human gut and bovine rumen, it is likely that relative abundance of a large number of species contributes to complex traits [2]. This is analogous to the many genes of small effect that contribute to complex traits, including disease [27] and BMI [28] in humans, and traits such as feed conversion efficiency in cattle [29], when the host genome is analysed. For such traits, individual host DNA markers explain only a minute fraction of the phenotypic variance. However, genomic predictions based on large numbers of genome wide DNA markers have been used to accurately predict future phenotypes [30].

Inspired by this encompassing statistical approach, we propose that metagenomic profiles [21] can be used to predict complex microbiome associated traits.

## Results

We have used metagenomic profiles to predict phenotypes in humans and cattle, and subsequently tested the accuracy of these predictions. A metagenomic profile is the relative abundance of several thousand microbial species from a metagenomic sample derived from untargeted massively parallel sequencing (MPS). In practice, all species in a microbiome cannot currently be identified and fully sequenced, so counts of sequence reads from samples aligning to contigs from metagenomic databases can be used. Metagenomic profiles for a group of samples are defined as an *n* × *m* matrix **X** with elements x_ij_, the log transformed and standardised count for sample *i* for contig *j*, with *n* samples and *m* contigs. The relationship between samples can then be described by a matrix **G** = **XX’/** *m*. Metagenomic profiles have previously been used to assess the relationship between samples [21], here their use is significantly expanded to predict phenotypes from the metagenomic relationship matrix using genomic best linear unbiased prediction (BLUP) [31]. The metagenomic predictions require a reference population of individuals with both the target phenotype and quantitative metagenomic profiles. Subsequently, future phenotypes can be predicted for any individual from their metagenomic profiles alone. To determine the prediction accuracy of such a method phenotypes can be predicted on a subset of records which have measured phenotypes that were not included in the model. The predicted phenotypes can then be correlated with the observed (real) phenotypes to give the accuracy of prediction (Pearson’s correlation coefficient r).

To test the utility of metagenomic predictions this method was used to first predict IBD status from 38 human faecal metagenomes from Spain [8]. The “case” individuals were in clinical remission from IBD. IBD is an inflammatory disorder of the digestive tract and includes diseases such as ulcerative colitis and Crohn’s disease. We were able to predict disease state in this dataset with an average accuracy of 0.423±0.091 from the faecal metagenomic profiles (Table 1) using three fold cross validation (the individuals being predicted were *always* omitted from the reference or training data set). When the predictions were converted to a most likely phenotype (prediction values >0.5 = normal, prediction value <0.05 = case), the correct phenotype was assigned 73.7% of the time. This is greater than the null predictor (predicting the most common phenotype every time) of 65.8% correct. The area under the receiver operator characteristic curve (AUC) was 0.76, calculated using [32].

**Table 1:** Metagenomic predictions of qualitative and quantitative traits.

Trait	Validation Method	Ref.Pop. (N)	Val. Pop. (N)	Accuracy (r)	95% CI #	Significant
IBD*	3-fold CV	Spain (25+13)	Spain (25+13)	0.429	0.156∶ 0.647	Y
BMI	2-fold CV	Denmark (84)	Denmark (84)	0.391	0.175∶ 0.491	Y
BMI	2 Populations	Denmark (84)	Spain-c (13)	0.101	−0.228∶ 0.624	N
Methane	2 Populations	bovGMC (31)	bovFT-t (8)	0.788	0.132∶0.961	Y
Methane	2 Populations	bovGMC (31)	bovFT-c (7)	0.404	0.330∶0.985	Y
Methane	2 Populations	bovGMC (31)	bovFCE (16)	0.466	0.165∶0.734	Y
Methane	2 Populations	bovFT (15)	bovFCE (16)	0.394	0.078∶0.711	Y
Methane	2 Populations	bovFT (15)	bovGMC-c (11)	−0.167	−0.677∶0.247	N
Methane	2 Populations	bovFT (15)	bovGMC-t (20)	0.277	0.347∶0.735	Y
Methane	2 Populations	bovFCE (16)	bovFT-t (8)	0.285	−0.283∶0.872	N
Methane	2 Populations	bovFCE (16)	bovFT-c (7)	0.780	0.127∶0.973	Y
Methane	2 Populations	bovFCE (16)	bovGMC-c (11)	0.084	−0.283∶0.528	N
Methane	2 Populations	bovFCE (16)	bovGMC-t (20)	0.376	0.049∶0.730	Y

Accuracy of prediction with confidence intervals for human and bovine metagenomic predictions. Phenotypes were predicted from metagenomic profiles using BLUP, performed in ASReml. To evaluate the accuracy of metagenomic predictions the predicted phenotype was correlated with the measured (real) phenotype. IBD and BMI data is from [8].

# 95% confidence interval of the Pearson’s correlation coefficient r based on 10,000 bootstraps.

Ref.Pop. = Reference population.

Val.Pop = Validation population.

Spain-c = Control samples from Spain (no IBD).

bovFT-t/bovGMC-t = Animals on the treatment diet only.

bovFT-c/bovGMC-c = Animals on the control diet only.

N = total number of samples used.

CV = Cross Validation.

2 Populations = Validation on a second independent population.

* Phenotypes used were IBD = 0, nonIBD = 1.

Following the success of predicting this qualitative trait we used the same method to predict BMI in humans, a continuous complex trait with significant implications for health. Because of the large number of samples (84) with reasonably complete phenotype data, we again used the Denmark metagenome dataset [8] to predict BMI. We were able to predict BMI in these samples with an average accuracy of 0.422±0.031 (Table 1) using two fold cross validation (Figure S2 in File S1). We then attempted to use the Denmark data as a reference set to predict BMI in the Spanish data. The correlation between our predictions and the measured phenotypes was again positive, but much lower (r = 0.101; Table 1), likely a reflection of significant microbial profile by environment interaction.

The final complex trait we applied the method to was methane emission levels from dairy cattle. Methane is a potent greenhouse gas which is produced through enteric fermentation of ruminants such as cattle (*Bos taurus*), and interestingly is also an indicator of microbial metabolic functions associated with disease states in humans [33]. Individual measurements of methane production from cattle are currently expensive and time consuming. For industry wide selection of animals for reduced methane emission levels more cost effective indicator phenotypes that can be measured on individual animals are required. We generated a dataset of *B. taurus* rumen metagenome samples [34] that had associated methane production values from three experiments (Table S1, Table S2 and Figure S3 in File S1). The first experiment, referred to here as bovGMC, had 31 animals randomly split between methane mitigating feed additive (grapemarc, which is a by-products of the wine industry composed of the remaining skin and seed residue of the grapes after the juice has been extracted) and control diets. The second experiment, referred to as bovFT, was a crossover design with 8 animals, who were each fed a control diet, alternated with a diet which had added methane mitigating feed additives (lipids from cottonseed and tannins from *Acacia mearnsii*). The third experiment, referred to as bovFCE, contained animals all fed a more typical industry diet (lucerne cubes and crushed wheat). The phenotypes in bovFCE reflect natural variation in methane emissions. We have used methane corrected for dry matter intake as the phenotype for the following predictions.

We predicted methane production using each of the three bovine datasets as the reference population, and predicted the phenotypes of animals in the other two datasets. The accuracy of these predictions was 0.553±0.119, 0.163±0.171 and 0.381±0.146 for the bovGMC, bovFT and bovFCE reference datasets respectively (Table 1). Predictions using the bovGMC dataset as the reference population outperformed both the bovFT and bovFCE reference datasets, with all bovGMC based predictions being significantly different to zero (based on 95% confidence interval from bootstrapping; Table 1). This may suggest that inclusion of bovGMC in the training set is important for these predictions; perhaps because these animals have extreme phenotypes, that is, the treatment diets lowered methane production (in grams per Kg dry matter intake) by 20% (Table S1 in File S1), thereby giving the predictions more power. As the treatment diet of bovFT lowered methane production by only 6% (Table S1 in File S1) it likely provided less predictive power then bovGMC. Another possibility is a reference population size effect, as bovGMC had double the number of animals in the other bovine datasets. Larger training datasets, particularly those with extreme phenotypes, may allow these prediction accuracies to be increased.

**Figure 1:**
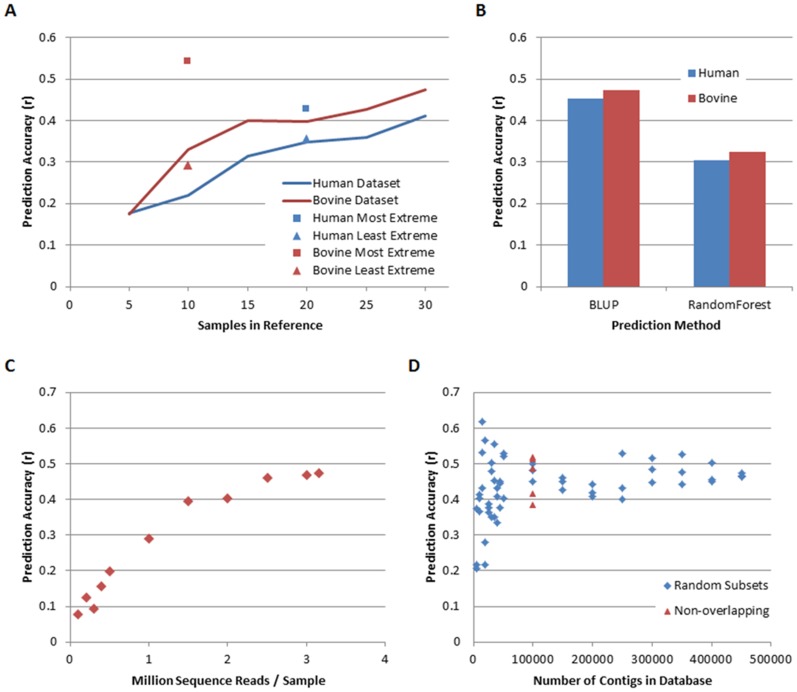
Reference population characteristics effect on metagenomic prediction accuracy. Prediction of residual enteric methane production from cattle (Red in panels a-c), and body mass index (BMI) from humans (Blue in panels a-c). Bovine predictions all use bovGMC as the reference population and bovFCE as the validation population. A) Lines: effect of reference population size on prediction accuracy. Line indicates the average accuracy of prediction from 20 random replicate populations sampled from the whole dataset. Squares: Accuracy of prediction when the most extreme phenotypes were used in the reference. Triangles: Accuracy of prediction when least extreme samples were used in the reference. B) Comparison of prediction accuracy using the BLUP and randomForests methods. The same reference and validation populations were used in the BLUP and randomForest methods. The randomForest predictions were performed with default settings, and the average correlation of 100 replicate runs is reported. C) Prediction accuracies under different sequence depths in the bovine dataset, phenotype is residual methane production, reference population is bovGMC, validation population is bovFCE. D) Prediction accuracy when different sized contig databases were used. Phenotype is residual methane production, reference population is bovGMC, and validation population is bovFCE. Blue diamonds: N contigs were randomly selected from the whole dataset. Red triangles: Contigs were randomly assigned to 4 groups of 100,000 contigs (no overlap between contig groups).

We next tested the effect of 1) reference size and 2) extent of reference phenotype variation, on predictive power of this method. To ensure the accuracies were directly comparable we used the bovGMC to predict the phenotypes of bovFCE and half of the Denmark BMI data to predict the other half in all of the following tests. To examine the effect of reference population size we randomly sampled animals from both the bovine and human reference population to generate 20 replicates of ‘sub’-reference populations of varying sizes. As the number of samples in the reference population increased the accuracy of prediction also increased (Figure 1a). Further, to test the hypothesis that having extreme phenotypes in the reference resulted in more accurate predictions we divided the reference into groups based on how extreme their phenotype was. For the bovine dataset, the reference population with the most extreme phenotypes predicted the validation set with the highest accuracy (Figure 1a; r = 0.541, 95% CI: 0.391 to 0.813), while the least extreme reference population resulted in prediction accuracies that were not significantly different to 0 (95% CI least extreme −0.121 to 0.597). A similar, but less extreme, pattern was observed when the human BMI reference dataset was divided into the 20 most (10 highest and 10 lowest) and 20 least (20 closest to the median) extreme BMI phenotypes. The prediction accuracy was higher when the 20 most extreme phenotypes were used as the reference dataset (most extreme BMI: r = 0.427, 95% CI 0.185 to 0.634; least extreme BMI: r = 0.35, 95% CI 0.093 to 0.533; Figure 1a).

The amount of sequencing required to utilise this method is of key importance as it directly affects the cost per sample. There are two independent sequencing events for this method, firstly the sequencing depth of each sample, and secondly the contig database production. We tested the effect of these two parameters on the accuracy of prediction in the bovine dataset, again using bovGMC to predict bovFCE, with residual methane production as the phenotype. Prediction accuracy increased as the sequence depth of samples increased, and began to plateau at approximately 2.5 million reads per sample (Figure 1c). We then tested the effect of the contig database size by randomly selecting N contigs from the **X** matrix. When the number of contigs used was small, there was a large amount of variation in the prediction accuracies observed, this variation decreased as the number of contigs used (N) was increased. However, as N increased there would be more overlap between the replicates, hence subsets with large N would be more similar to each other than subsets with small N, this could possibly explain the reduction in prediction accuracy variation. To test if the lower prediction accuracy variation was due to overlap between the random contig subsets: contigs were randomly assigned to four non-overlapping subsets of 100,000 contigs per subset. The variation between these subsets was lower than the variation at smaller Ns (Figure 1d).

There are other possible methods which could be used to quantify ‘raw’ metagenomic data. One such strategy is K-mer counts in place of alignment counts. This would have the advantage of using the whole dataset, while alignments are only able to use information from reads which align to the reference (Table S3 in File S1). To test the effect of using K-mers we made the *n* × *m* metagenomic relationship matrix **X** with elements x_ij,_ the log transformed and standardised count for sample *i* for K-mer *j*, with *n* samples and *m* K-mers. Only K-mers that were observed in every sample were used. We used K = 31 and K = 11. The same model as used for the contig count matrix was then applied to this K-mer matrix. This method was tested on the bovine datasets (reference = bovGMC, validation = bovFCE), with residual methane production as the phenotype. The K-mer based prediction accuracy was not significantly different to 0 (K = 11: r = 0.126, 95% CI: −0.136 to 0.587; K = 31: r = 0.018, 95% CI: −0.278 to 0.562). Using K-mers performs poorly compared to using read counts.

Other predictive models have been applied to 16S metagenome data. Knights et al. [35] identified that randomForests performed the best out of a number of different methods based on several 16S metagenomic datasets (not shotgun MPS data). To compare metagenomic predictions using BLUP with the randomForests approach we applied randomForests [36] to the bovine and human datasets. The randomForests method gave accuracies of r = 0.305 and 0.325 for the human and bovine datasets respectively. These accuracies were both lower than those observed using BLUP (Figure 1b).

In genome wide association studies, SNP are fitted one at a time. The analogy in our data sets would be to test each contig in turn (where the X variable is the number of sequence reads mapping to the contig for each individual, rather than SNP genotypes). We applied this approach to our bovine dataset. There was a very low validation rate for the most significant contigs. We achieved a maximum r of 0.19 when using multiple contigs to predict methane production (Figure S4 in File S1).

## Discussion

BLUP has been used in animal breeding studies for several years (for examples see [37]–[40]), however it has never before been applied to metagenomic profiles of untargeted shot-gun sequence reads. In this dataset we have found metagenomic predictions using BLUP more accurate than using either individual contigs, a subset of the most significant contigs or the randomForest algorithm. Additionally, BLUP is not computationally demanding, which will allow future studies to include large numbers of samples without computational limitation. With recent technological developments, obtaining 3 million reads per sample is technically straightforward and provides a good representative sample of the rumen microbiome [21]. For the accuracies of metagenomic predictions to improve more metagenomic datasets with well characterised phenotypes need to be accumulated.

We observed that the accuracy of prediction increased with increasing numbers of individuals in the reference population, however the numbers used in the current study were not great enough for this increase to plateau, therefore an estimate of the number of samples required to achieve maximum accuracy (in proportion to the amount of variation explained by the microbiome) predictions cannot be made at this stage. We also observed that having extreme phenotypes in the reference population gives more accurate predictions, although to a much greater extent in the bovine dataset than the human dataset. Additionally it appears, from observations of the bovine dataset, that both the read depth and contig database size used here is adequate to enable prediction accuracies to stabilise. Therefore, at this stage it is advisable for studies using metagenomic predictions to aim for three million sequence reads per sample, additionally we recommend that reference populations should be larger than those used here if obtainable, and should contain individuals with extreme phenotypes where possible (although this may have stronger influences on some traits than others).

Metagenomic profiles that are based on alignments to contigs appear to dramatically outperform profiles based on K-mer counts in terms of their ability to predict phenotypes, despite the fact that using K-mers allows the entire dataset to be utilised while alignments to contigs only uses a proportion of the data. The advantage of the contig based method is that the sequencing does not have to be deep enough to have overlapping fragments, as all reads that map to a contig are grouped. In contrast, with the K-mers approach, the same K-mers may be observed in a large number of species, hence the K-mers profile does not reflect species abundances. The percentage of reads mapping does not seem to inhibit the metagenomic predictions. The human dataset had a much larger percentage of reads aligning to the reference than the bovine datasets (Table S3 in File S1), however BMI was not predicted more accurately than enteric methane production. The random forest method tested performed reasonably, but not as well as BLUP. Therefore the underlying assumption of BLUP (read counts at all contigs are associated with a small, but non zero effect on the phenotype), appears to be a valid assumption for this type of data.

We have used metagenomic predictions to predict phenotypes from multiple traits and in two different species. Development of metagenomic predictions may have wide reaching applications, including aiding in diagnosis of digestive tract disorders, and applications in reducing green-house gas production from agriculture, as well as other microbiome associated traits. Although the BMI trait used here is easily measured directly, accurately measuring enteric methane production from cattle is expensive. If the prediction accuracy of enteric methane production level could be increased it is possible that metagenomic predictions could aid efforts to reduce greenhouse gas production from agriculture. The datasets currently available are not substantial enough for these applications to be realised, however achieving significant predictions with such small population sizes bodes well for the potential of metagenomic predictions in the future. The AUC for GWAS-based predictions for IBD is approximately between 0.65 and 0.75 [41]. Our results show that microbiome based predictions for IBD have an AUC value at the higher end of this range (0.76). The vastly different sample sizes (thousands of individuals are included in GWAS studies and only up to 38 individuals in our microbiome predictions) makes it difficult to compare the effectiveness or complementarity of genome based and microbiome based predictions. The fact that we can achieve similar accuracies with much smaller sample size only reinforces the potential for metagenomic profile predictions.

A recent study [9] performed a metagenome-wide association study to identify aspects of the human microbiome associated with type 2 diabetes. They observed a significant correlation between their predicted and real phenotypes. The success of their metagenome-wide association study, with a small number of selected predictors, compared to our prediction using all contig counts simultaneously, may be related to the size of the training dataset, which was much smaller in this study than in Qin et al. [9], or may be due to the different architechtures of effect of metagenome profile on the different traits in the studies. Metagenome-wide association studies have the advantage of identifying the species driving the phenotype, however if the phenotype is due to small effects from many species then their power would be more limited. Therefore the purpose of the study must be considered, metagenomic prediction will likely produce more accurate phenotype predictions, however if intervention is the desired result then metagenome-wide association studies will provide targets that may be manipulated.

The disease state predictions have scope to be more accurate as the phenotype approaches quantitative measurements (i.e. the level of phenotyping is at greater resolution than affected/not affected). Differences between the gut microflora of clinically remissive IBD patients and non-IBD controls may be expected to be quite subtle, however multiple studies have found clear differences between the microbiomes of the IBD versus control patients’ samples using principal component analysis [8] and metabolic network [25] approaches. It is clear that there are differences in microbiomes between IBD and non-IBD patients, however as with many trait associated metagenomic changes, it is not yet clear whether these changes are a cause or result of IBD status [33].

Genomic predictions from a host’s own genome may be limited in that variation in the microbiome cannot be taken into account. This may be particularly relevant when considering diseases or phenotypes that are closely linked to the microorganisms residing within the host, such as bowel disorders or enteric greenhouse gas production. The method described here for predicting complex phenotypes from metagenome profiles could be combined with genomic predictions from the host’s own DNA to maximise accuracy of predicting phenotypes. The achievable accuracy of prediction for traits such as methane production is thus far unknown, however the best possible model would likely include metagenomic predictions, genomic predictions and perhaps physiological trait measures (such as body weight to account for rumen volume). Larger numbers of animals are required to accurately test such a model.

One clear limitation of metagenomic predictions compared to genomic predictions is that the microbiome of the host is variable, that is, it may change in response to diet or other environmental factors over time, whereas the hosts DNA remains constant. More research is required to investigate the stability of microbiome profiles over time; however, the results indicate that this approach may be informative for clinical trials and genetic studies where the microbiome is expected to be involved. The interaction between metagenomic predictions (predictions based on the microbiome) and genomic predictions (predictions based on the host phenotype) of these traits also warrants investigation. More immediately, the approach developed here appears accurate enough to be valuable as surrogate phenotypes for traits that are difficult to measure, such as enteric methane production.

## Methods

### Ethics Statement

All work involving animals was approved by the Department of Environment and Primary Industries Agricultural Research & Extension Animal Ethics Committee.

### Bovine Rumen Dataset

Three bovine datasets were used in this study: bovGMC, bovFT, bovFCE. All animals were located at Department of Environment and Primary Industries Ellinbank Centre, Victoria, Australia. The data was also used in [34].

The bovGMC experiment is described in more detail in Moate et al. [42], which describes the parent experiment that samples were taken from. Breifly, bovGMC had cows randomly assigned to either a control diet approximately 4.0 kg dry matter (DM) of crushed wheat, 0.2 kg DM of molasses, 0.1 kg DM of minerals and 14 kg DM of lucerne hay, or a treatment diet which in consisted of the control diet with 5 kg DM/d of either dry crumbled or ensiled grape marc substituted for an equal quantity of alfalfa hay. These diets were fed for two weeks prior to methane measurement via the SF_6_ technique [43] and rumen sample collection via stomach pump. The animals on treatment diets had 19.8% less methane production than the animals on the control diets (controls: 25.75 gCH_4_/KgDMI; treatments: 20.66 gCH_4_/KgDMI; t-test, p<0.0001).

The bovFT experiment was a crossover design with 8 fistulated animals. Animals were fed the treatment or control diet for two weeks prior to methane measurement over two days via respiration chamber (as described in [44]) and rumen sampling via the fistulae. In addition to the base diet of 6.0 kg DM of concentrates (4.1 kg DM crushed wheat; 1.5 kg DM cold pressed canola meal; 0.12 kg DM mineral mix and 0.28 kg DM palabind molasses powder) and *ad libitum* lucerne hay, the treatment diet of the FT study had 800 g of cottonseed oil and 400 g of raw tannin from *Acacia mearnsii* added to the rumen though the fistulae each day. The control group in the FT experiment had 800 mL of water added though the fistulae every day. The animals on treatment diet had 6.26% less methane production than the animals on the control diets (controls: 21.64 gCH_4_/KgDMI; treatments: 20.29 gCH_4_/KgDMI; paired t-test, p = 0.012).

The bovFCE group were all fed a control diet 6 kg DM crushed wheat per cow per day plus compressed cubes of lucerne hay offered *ad libitum*. The diet was fed for a minimum of two weeks prior to methane production being measured via respiration chambers (as described in [44]) and rumen fluid collection via stomach pump. These samples were taken from a larger study investigating feed conversion efficiency [45].

Rumen fluid samples were taken from animals within 6 hours of methane measurements being completed (Table S1 in File S1). DNA was extracted using the PowerMax Soil DNA Isolation kit (MoBio) and sequenced on the HiSeq2000 (Illumina) as per Ross et al. [21]. After sequencing the whole genomic DNA, poor quality sequence was removed from the dataset. Bases from the 3′ end of the read were removed until there was a maximum of 3 bases remaining in the read with a Phred quality score of <15. If the read was <50 bp long after this trimming, or the average Phred quality of the read was <30 it was discarded. The remaining data was decloned using ‘*kmers_remove_clonal*’ from libngs (https://github.com/sylvainforet/libngs).

The contig database used for the bovine predictions was the combined contigs from Hess et al. [19] and Ross et al. [21]. The bovine metagenome data used in this study can be freely obtained from MG-RAST [35] project ID: 4126 (Bovine Metagenome).

### Methane Phenotype

The methane phenotype used in the metagenomic predictions was methane production corrected for dry matter intake. This was done by taking the residuals of a linear model fitting dry matter intake to methane production. Where data from multiple days was available the phenotype was averaged of both days.

### Other Sequences

Human metagenome sequences were obtained from Qin et al. [8]. This data was used for the IBD and BMI predictions. The database used for the human BMI and IBD predictions consisted of 4 Gb faecal derived contigs from http://www.hmpdacc.org/HMASM/.

### Metagenomic Predictions

Here we have used metagenomic profiles [21] to create metagenomic relationship matrices (Figure S1 and Figure S5 in File S1). A metagenomic profile is the vector of counts of MPS shotgun reads that align to each contig in a database (we used BWA-backtrack to perform alignments [46], with the exception of the –e0 and –o0 flags [allowing no gaps] in the ‘aln’ command all other parameters were left as default). Metagenomic profiles relate to the relative (not absolute) abundance of the microbial markers. As the model used here assumes a normal distribution the metagenomic profiles were log transformed and standardised. Several metagenomic profiles were combined form an *n* × *m* matrix **X** with elements x_ij_, the log transformed and standardised count for sample *i* for contig *j*, with *n* samples and *m* contigs. Contigs with <10 reads in total aligning to them were removed from the matrix prior to standardising. These profiles were then compared to make a rumen microbiome relationship matrix (calculated as **G** = **XX’/** *m*). Best linear unbiased prediction [31] was then used to predict phenotypes for validation samples. A mixed model was fitted to the data: **y** = **1**_n_ *µ*+**Zg**+**e**. Where y is the a vector of phenotypes, with one record per sample, **1**_n_ is a vector of ones, *μ* is the overall mean, **Z** is a design matrix allocating records to samples, and g is a random effect estimate ∼ N (0, **G**σ^2^_g_ ). Using ASReml [47], σ^2^_g_ was estimated from the data and the phenotypes of the samples (ĝ which is a vector of length *n*) were predicted as:
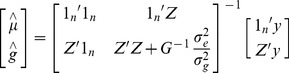



Solving the equations results in an estimate of the mean and an estimate of the residual for each metagenome profile, such that ĝ has the dimensions *n* × 1. For each metagenome profile, the predicted phenotype is ĝ_i_+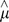.

Instructions for running metagenomic predictions are presented in File S1. A script implementing metagenomic predictions using the free R statistical package and rrBLUP [48], along with some small example files is available in File S2.

### Accuracy Assessment

For both qualitative and quantitative traits, the accuracy is determined by Pearson’s ‘r’, that is, the correlation between the observed and predicted phenotype. An accuracy of 0 or less indicates the predictions are no better than chance (this is true for both qualitative and quantitative applications). An accuracy of 1 would imply perfect concordance between observed and predicted phenotypes, and 100% certainty for qualitative trait assignments. For qualitative traits the accuracy can also be interpreted as the percentage of correct phenotypes when the predictive value is turned into a ‘most likely’ phenotype class.

### Cross Validation

Three way cross validation was performed for the IBD predictions, when there were not two independent datasets available for validation. Data was split into three equally sized groups. Each group was predicted from the other two groups in turn, such that A was predicted from B+C; B predicted from A+C and C predicted from A+B. Each sample was predicted once. Each sample was used as a reference twice. Results were collated to achieve the final correlation between predicted and measured phenotype. Individual group correlations for IBD were 0.259, 0.438 and 0.574 for groups A, B and C respectively.

Two way cross validation was performed for the BMI predictions. The Danish dataset was split into two groups. Group A was predicted using group B as a training set; Group B was predicted using group A as a training set. Individual group correlations for BMI were 0.453 and 0.391 for groups A and B respectively.

### 95% Confidence Intervals

95% confidence intervals (CI) for the Pearson’s correlation coefficient were calculated by bootstrapping. 10,000 bootstraps were preformed per metagenomic prediction. A new dataset of equal size was sampled from the original correlations with replacement. The correlation was then recalculated. This was repeated 10,000 times. The 95% CI was extracted from this data.

### K-mer Based Prediction

Single reads that passed trimming with a length of 100 bp from the bovine dataset 1 and bovine dataset 3 were used for the K-mer analysis. Every observed K-mer in one million reads per sample were counted using jellyfish [49]. The counts were then formed into a matrix of sample x K-mer. Metagenomic predictions were then performed as per the contig alignment based method described above, including removal of K-mers with <10 counts and standardisation of the matrix.

### Reference Population Effects

The same validation and reference populations were used in all reference population size tests and the extreme phenotype tests, as well as the K-mer based prediction described above. To test the effect of reference population size: N individuals were randomly selected from the reference population, metagenomic predictions were performed using BLUP as described above. This was repeated 20 times for each value of N. To test the effect of extreme phenotypes the 5 (bovine) and 10 (human) samples with the highest and lowest phenotypic values were used in the ‘most extreme’ reference dataset. The ‘least extreme’ reference dataset consisted of the animals which, when ranked according to phenotype, were in the middle of the dataset. Metagenomic predictions were performed, using these most and least extreme reference datasets, as described above.

### CH_4_ Prediction by Contig

A regression analysis was performed to examine if the effect of each contig could be used to predict methane production from the bovine dataset. A linear model was fitted to each contig (after filtering to remove contigs with <100 reads mapping in the reference or the validation population). Contigs were then ranked by significance of the model. The model results were then used to predict methane production in the validation population (Figure S4 in File S1). Additionally, the predicted methane production was also averaged over a number of contigs.

More significant contigs in the reference population were not any better at predicting methane production in the validation population than less significant contigs. When the average of multiple contig predictions was used, the ability to predict methane production in the validation population, to a maximum of r^2^ = 0.04 (r = 0.19; 95% CI_r_ = 0.102–0.665; Figure S4 in File S1) when the most significant 205 contigs were included.

## Supporting Information

File S1 **Contains Tables S1, S2 and S3, Figures S1, S2, S3, S4 and S5, instructions for running metagenomic predictions and calculations of the number of prokaryote cells in the bovine rumen.**
(PDF)

File S2 **Contains scripts for running metagenomic predictions (MetagenomicPredictionForASReml.R, MetagenomicPredictions.R), an example metagenomic profile matrix (MPM.txt) and an example phenotype file (Phenotypes.txt).**
(ZIP)

## References

[1] Dethlefsen L, McFall-Ngai M, Relman DA (2007) An ecological and evolutionary perspective on human-microbe mutualism and disease. Nature 449: 811–818. 17943117 10.1038/nature06245 PMC9464033

[2] Cho I, Blaser MJ (2012) The human microbiome: at the interface of health and disease. Nature Reviews Genetics 13: 260–270. 10.1038/nrg3182 PMC3418802 22411464

[3] Savage DC (1977) Microbial ecology of the gastrointestinal tract. Annual Review of Microbiology 31: 107–133. 10.1146/annurev.mi.31.100177.000543 334036

[4] Segata N, Haake SK, Mannon P, Lemon KP, Waldron L, et al (2012) Composition of the adult digestive tract bacterial microbiome based on seven mouth surfaces, tonsils, throat and stool samples. Genome Biology 13: R42. 22698087 10.1186/gb-2012-13-6-r42 PMC3446314

[5] Li K, Bihan M, Yooseph S, Methe BA (2012) Analyses of the Microbial Diversity across the Human Microbiome. PLoS One 7: e32118. 22719823 10.1371/journal.pone.0032118 PMC3374608

[6] Turnbaugh PJ, Ley RE, Hamady M, Fraser-Liggett CM, Knight R, et al (2007) The human microbiome project. Nature 449: 804–810. 17943116 10.1038/nature06244 PMC3709439

[7] Koren O, Goodrich JK, Cullender TC, Spor A, Laitinen K, et al (2012) Host Remodeling of the Gut Microbiome and Metabolic Changes during Pregnancy. Cell 150: 470–480. 22863002 10.1016/j.cell.2012.07.008 PMC3505857

[8] Qin J, Li R, Raes J, Arumugam M, Burgdorf KS, et al (2010) A human gut microbial gene catalogue established by metagenomic sequencing. Nature 464: 59–65. 20203603 10.1038/nature08821 PMC3779803

[9] Qin J, Li Y, Cai Z, Li S, Zhu J, et al (2012) A metagenome-wide association study of gut microbiota in type 2 diabetes. Nature 490: 55–60. 23023125 10.1038/nature11450

[10] Lavery T, Be R, Seymour J, Mitchell J, Jeffries T (2012) High nutrient transport and cycling potential revealed in the microbial metagenome of Australian sea lion (Neophoca cinerea) Faeces. 10.1371/journal.pone.0036478 PMC3350522 22606263

[11] Alcaide M, Messina E, Richter M, Bargiela R, Peplies J, et al (2012) Gene Sets for Utilization of Primary and Secondary Nutrition Supplies in the Distal Gut of Endangered Iberian Lynx. PLoS One 7: e51521. 23251564 10.1371/journal.pone.0051521 PMC3520844

[12] Barry KA, Middelbos IS, Vester Boler BM, Dowd SE, Suchodolski JS, et al (2012) Effects of dietary fiber on the feline gastrointestinal metagenome. Journal of proteome research 11: 5924–5933. 23075436 10.1021/pr3006809

[13] Swanson KS, Dowd SE, Suchodolski JS, Middelbos IS, Vester BM, et al (2011) Phylogenetic and gene-centric metagenomics of the canine intestinal microbiome reveals similarities with humans and mice. ISME Journal 5: 639–649. 20962874 10.1038/ismej.2010.162 PMC3105739

[14] Lamendella R, Santo Domingo JW, Ghosh S, Martinson J, Oerther DB (2011) Comparative fecal metagenomics unveils unique functional capacity of the swine gut. BMC microbiology 11: 103. 21575148 10.1186/1471-2180-11-103 PMC3123192

[15] Xu B, Xu W, Yang F, Li J, Yang Y, et al (2013) Metagenomic Analysis of the Pygmy Loris Fecal Microbiome Reveals Unique Functional Capacity Related to Metabolism of Aromatic Compounds. PLoS One 8: e56565. 23457582 10.1371/journal.pone.0056565 PMC3574064

[16] Durso LM, Harhay GP, Bono JL, Smith TPL (2011) Virulence-associated and antibiotic resistance genes of microbial populations in cattle feces analyzed using a metagenomic approach. Journal of microbiological methods 84: 278–282. 21167876 10.1016/j.mimet.2010.12.008

[17] Hildebrand F, Ebersbach T, Nielsen H, Li X, Sonne S, et al (2012) A comparative analysis of the intestinal metagenomes present in guinea pigs (Cavia porcellus) and humans (Homo sapiens). BMC genomics 13: 514. 23020652 10.1186/1471-2164-13-514 PMC3472315

[18] Dai X, Zhu Y, Luo Y, Song L, Liu D, et al (2012) Metagenomic Insights into the Fibrolytic Microbiome in Yak Rumen. PLoS One 7: e40430. 22808161 10.1371/journal.pone.0040430 PMC3396655

[19] Hess M, Sczyrba A, Egan R, Kim T-W, Chokhawala H, et al (2011) Metagenomic discovery of biomass-degrading genes and genomes from cow rumen. Science 331: 463–467. 21273488 10.1126/science.1200387

[20] Brulc JM, Antonopoulos DA, Berg Miller ME, Wilson MK, Yannarell AC, et al (2009) Gene-centric metagenomics of the fiber-adherent bovine rumen microbiome reveals forage specific glycoside hydrolases. Proceedings of the National Academy of Sciences of the United States of America 106: 1948–1953. 19181843 10.1073/pnas.0806191105 PMC2633212

[21] Ross EM, Moate PJ, Bath CR, Davidson SE, Sawbridge TI, et al (2012) High throughput whole rumen metagenome profiling using untargeted massively parallel sequencing. BMC Genetics 13: 53. 22747657 10.1186/1471-2156-13-53 PMC3464612

[22] Singh K, Ahir V, Tripathi A, Ramani U, Sajnani M, et al (2012) Metagenomic analysis of Surti buffalo (*Bubalus bubalis*) rumen: a preliminary study. Molecular Biology Reports 39: 4841–4848. 21947953 10.1007/s11033-011-1278-0

[23] Turnbaugh PJ, Hamady M, Yatsunenko T, Cantarel BL, Duncan A, et al (2009) A core gut microbiome in obese and lean twins. Nature 457: 480–484. 19043404 10.1038/nature07540 PMC2677729

[24] Yatsunenko T, Rey FE, Manary MJ, Trehan I, Dominguez-Bello MG, et al (2012) Human gut microbiome viewed across age and geography. Nature 486: 222–227. 22699611 10.1038/nature11053 PMC3376388

[25] Greenblum S, Turnbaugh PJ, Borenstein E (2012) Metagenomic systems biology of the human gut microbiome reveals topological shifts associated with obesity and inflammatory bowel disease. Proceedings of the National Academy of Sciences 109: 594–599. 10.1073/pnas.1116053109 PMC3258644 22184244

[26] Papa E, Docktor M, Smillie C, Weber S, Preheim SP, et al (2012) Non-invasive mapping of the gastrointestinal microbiota identifies children with inflammatory bowel disease. PLoS One 7: e39242. 22768065 10.1371/journal.pone.0039242 PMC3387146

[27] Hirschhorn JN, Gajdos ZK (2011) Genome-wide association studies: results from the first few years and potential implications for clinical medicine. Annual Review of Medicine 62: 11–24. 10.1146/annurev.med.091708.162036 21226609

[28] Yang J, Ferreira T, Morris AP, Medland SE, Genetic Investigation of ATC, et al.. (2012) Conditional and joint multiple-SNP analysis of GWAS summary statistics identifies additional variants influencing complex traits. Nature Genetics 44: 369–375, S361–363. 10.1038/ng.2213 PMC3593158 22426310

[29] Pryce JE, Arias J, Bowman PJ, Davis SR, Macdonald KA, et al (2012) Accuracy of genomic predictions of residual feed intake and 250-day body weight in growing heifers using 625,000 single nucleotide polymorphism markers. Journal of Dairy Science 95: 2108–2119. 22459856 10.3168/jds.2011-4628

[30] Meuwissen T, Hayes B, Goddard M (2001) Prediction of total genetic value using genome-wide dense marker maps. Genetics 157: 1819. 11290733 10.1093/genetics/157.4.1819 PMC1461589

[31] Henderson CR (1984) Applications of linear models in animal breeding. Guelph, Canada: University of Guelph Press.

[32] Sing T, Sander O, Beerenwinkel N, Lengauer T (2005) ROCR: visualizing classifier performance in R. Bioinformatics. 21: 3940–3941. 10.1093/bioinformatics/bti623 16096348

[33] Roccarina D, Lauritano EC, Gabrielli M, Franceschi F, Ojetti V, et al (2010) The role of methane in intestinal diseases. American Journal of Gastroenterology 105: 1250–1256. 20216536 10.1038/ajg.2009.744

[34] Ross EM, Moate PJ, Marett L, Cocks BG, Hayes BJ (2013) Investigating the effect of two methane mitigating diets on the rumen microbiome using massively parallel sequencing. Journal of dairy science: doi: 10.3168/jds.2013–6766. 10.3168/jds.2013-6766 23871375

[35] Knights D, Costello EK, Knight R (2011) Supervised classification of human microbiota. FEMS Microbiology Reviews 35: 343–359. 21039646 10.1111/j.1574-6976.2010.00251.x

[36] Liaw A, Wiener M (2002) Classification and Regression by randomForest. R news 2: 18–22.

[37] Belonsky GM, Kennedy BW (1988) Selection on individual phenotype and best linear unbiased predictor of breeding value in a closed swine herd. Journal of Animal Science 66: 1124–1131. 3397339 10.2527/jas1988.6651124x

[38] Hayes B, Bowman P, Chamberlain A, Goddard M (2009) Invited review: Genomic selection in dairy cattle: Progress and challenges. Journal of dairy science 92: 433–443. 19164653 10.3168/jds.2008-1646

[39] Brito FV, Neto JB, Sargolzaei M, Cobuci JA, Schenkel FS (2011) Accuracy of genomic selection in simulated populations mimicking the extent of linkage disequilibrium in beef cattle. BMC genetics 12: 80. 21933416 10.1186/1471-2156-12-80 PMC3224120

[40] Daetwyler HD, Swan AA, van der Werf JH, Hayes BJ (2012) Accuracy of pedigree and genomic predictions of carcass and novel meat quality traits in multi-breed sheep data assessed by cross-validation. Genetics Selection Evolution 44: 1–11. 10.1186/1297-9686-44-33 PMC3506471 23146144

[41] Jostins L, Barrett JC (2011) Genetic risk prediction in complex disease. Human Molecular Genetics 20: R182–188. 21873261 10.1093/hmg/ddr378 PMC3179379

[42] Moate PJ, Williams SRO, Ribaux BE, Wales WJ (2012) Feeding grape marc to dairy cows suppresses methane emissions. The 5th Australasian Dairy Science Symposium. 454–455.

[43] Johnson K, Huyler M, Westberg H, Lamb B, Zimmerman P (1994) Measurement of methane emissions from ruminant livestock using a sulfur hexafluoride tracer technique. Environmental Science & Technology 28: 359–362. 22176184 10.1021/es00051a025

[44] Grainger C, Clarke T, McGinn SM, Auldist MJ, Beauchemin KA, et al (2007) Methane Emissions from Dairy Cows Measured Using the Sulfur Hexafluoride (SF_6_) Tracer and Chamber Techniques. Journal of Dairy Science 90: 2755–2766. 17517715 10.3168/jds.2006-697

[45] Pryce J, Marett L, Wales W, Williams Y, Hayes B (2012) Calves selected for divergence in feed conversion efficiency for growth also exhibit divergence in feed conversion efficiency in lactation. Proceedings of the australiasian daiy science symposium: 45–46.

[46] Li H, Durbin R (2009) Fast and accurate short read alignment with Burrows–Wheeler transform. Bioinformatics 25: 1754–1760. 19451168 10.1093/bioinformatics/btp324 PMC2705234

[47] Gilmour AR, Gogel BJ, Cullis BR, Thompson R (2006) ASReml User Guide Release 2.0. VSN International Ltd, Hemel Hempstead, HP1 1ES, UK.

[48] Endelman JB (2011) Ridge regression and other kernels for genomic selection with R package rrBLUP. The Plant Genome 4: 250–255.

[49] Marçais G, Kingsford C (2011) A fast, lock-free approach for efficient parallel counting of occurrences of k-mers. Bioinformatics 27: 764–770. 21217122 10.1093/bioinformatics/btr011 PMC3051319

