# Supplementary material for: Metagenomic Predictions: From Microbiome to Complex Health and Environmental Phenotypes in Humans and Cattle
Source: PLoS One. 2013 Sep 4;8(9):e73056. doi: 10.1371/journal.pone.0073056 (PMC3762846; doi:10.1371/journal.pone.0073056)
Supplement: File S1 — Contains Tables S1, S2 and S3, Figures S1, S2, S3, S4 and S5, instructions for running metagenomic predictions and calculations of the number of prokaryote cells in the bovine rumen. (PDF) [file pone.0073056.s001.pdf]

File S1.

Metagenomic Predictions: from microbiome to complex health and environmental phenotypes in humans and cattle

E.M. Ross[a,b,c,*], P.J. Moate[d], L. Marett[d], B.G. Cocks[a,b,c], B.J. Hayes[a,b,c]

[a] Biosciences Research Division, Department of Environment and Primary Industries, Bundoora, VIC 3083, Australia
[b] Dairy Futures Cooperative Research Centre, Bundoora, VIC, Australia
[c] La Trobe University, Bundoora, VIC, Australia
[d] Future Farming Systems Division, Department of Environment and Primary Industries, Ellinbank, VIC, Australia
[*]Corresponding Author: elizabeth.ross@depi.vic.gov.au

Key words: Metagenome, microbiome, prediction, rumen, methane, BLUP

This document contains supporting material including:

- Instructions for running metagenomic predictions
- Calculations for the estimate of the number of cells in the bovine rumen
- Table S1
- Table S2
- Table S3
- Figure S1
- Figure S2
- Figure S3
- Figure S4
- Figure S5

Running Metagenomic Predictions

A script which converts a metagenomic profile matrix and phenotypes to files that can be run as ASReml [1] has been supplied. As ASReml is not a free product we have also implemented the method using the free R package rrBLUP [2]. Both scripts and some small example data are supplied in File S2 .

The provided scripts were designed for a linux operating system. All systems are administered differentially and you should contact you system administrator to ensure you have access to the required files and programs.

The script is coded in the R statistical language [3], which is free software available for may platforms. The script requires a space delimited count matrix (Figure S1a), and a space delaminated phenotype table (Figure S1b) which contains the reference populations phenotypes. The sample names in the count matrix must match exactly with the sample names used in the phenotype table. The rrBLUP script requires a third variable, which is the name of the file which the output will be put in (e.g. Output.txt).

Once R is installed, the scripts can be opened and run one line at a time, or they can be run using a command.

To run the rrBLUP version, which is called MetagenomicPredictions.R, place the script in the current working directory. Then run one of:

1. Rscript MetagenomicPredictions.R MPM.txt Phenotypes.txt Outfile.txt
2. R CMD BATCH --slave "--args MPM.txt Phenotypes.txt Outfile.txt" MetagenomicPredictions.R

To run the ASReml version, which is called MetagenomicPredictionForASReml.R, place the script in the current working directory. Then run one of:

1. Rscript MetagenomicPredictionForASReml.R MPM.txt Phenotypes.txt
2. R CMD BATCH --slave "--args MPM.txt Phenotypes.txt" MetagenomicPredictionForASReml.R

In this example MPM.txt contains a metagenomic profile matrix, Phenotypes.txt contains the phenotype table. In the rrBLUP version, the predictions will be placed in a new file with the name specified in the third variable (in the example given that is Outfile.txt). In the ASReml version four output files are made (.as, .phen, .giv and a sample name key). These files are named using the original file names plus an extension. The .as file can then be used to run ASReml if it is installed on the system.

Note that in the example files given the MetagenomicPredictionForASReml.R script has difficulty solving the relationship matrix. To deal with this situation the script tries adding 0.1 to the diagonal of the matrix. If this fails it adds a further +1 to the diagonal of the matrix. If the matrix can still not be solved the script exits.

Bacterial Cells in the Bovine Rumen

This is a calculation based on the reported number of bacteria per mL of rumen fluid [4] and adhered to the plant material and the rumen volume, compared to the average cell weight[5] versus the average cow weight. The average weight of total rumen contents from eight lactating Holstein cows was 106Kg (calculated by removing the total rumen contents through a fistula). The average empty cow weight of the same animals was 521Kg. We used an estimate that the rumen contained 77% rumen fluid (81.6L) by weight [6]. Studies have suggested that 70% of rumen microbes are adhered to the fibre component of rumen contents [7]. Based on these numbers we have tried to estimate the number of bacterial cells in the bovine rumen as compared to the animals own mammalian cells.

Based on cells in the rumen fluid:
(10^{11} bacterial cells/mL×81,600 mL) / (521,000,000,000,000ng÷2.3ng/mammalian cell) =
8.16×10^{15} bacterial cells / 2.27×10^{14} mammalian cells =
35.94 times more bacterial cells than bovine cells in rumen fluid only.

Only 30% of rumen microbes are in the fluid, 70% are firmly attached to the fibre component [7]. Therefore:
8.16×10^{15} bacterial cells in fluid + 1.90×10^{16} bacterial cells in fibre fraction = 2.72×10^{16} bacterial cells
2.72×10^{16} bacterial cells / 2.27×10^{14} mammalian cells =
119.8 times more bacterial cells than bovine cells in the bovine rumen

Alternatively, if a 70kg human has 10^{13} cells [8], a 521kg cow should have 7.44×10^{13} cells (assuming the same cell density per Kg). Therefore:
2.72×10^{16} bacterial cells / 7.44×10^{13} mammalian cells =
365.6 times more bacterial cells than bovine cells

These values are very speculative and should be treated with caution. Without bacterial counts per mL or per gram from the same animals that were weighted it is not possible to get an accurate account of the number of bacterial cells in the rumen. Additionally there is no accurate way to access the number of cells in the host animals. Because of this we report the lower of the two whole rumen microbe estimates in the body of the paper. A more though estimate may include microbes in other sections of the gastrointestinal tract, as well as other body sites such as skin.

Table S1. Rumen sample phenotypes.

Cow ID	Group	Diet	CH_4 measurement technique	RF Collection	CH4 g/kgDMI[†]
2202	bovFT	Con_{FT}[#]	Chamber	Fistulae	22.46
2202	bovFT	FT	Chamber	Fistulae	20.37
3063	bovFT	FT	Chamber	Fistulae	21.81
3063	bovFT	Con_{FT}[#]	Chamber	Fistulae	22.07
6803	bovFT	FT	Chamber	Fistulae	19.75
6803	bovFT	Con_{FT}[#]	Chamber	Fistulae	21.59
6838	bovFT	FT	Chamber	Fistulae	19.52
6852	bovFT	Con_{FT}[#]	Chamber	Fistulae	19.01
6852	bovFT	FT	Chamber	Fistulae	18.40
6859	bovFT	Con_{FT}[#]	Chamber	Fistulae	21.99
6859	bovFT	FT	Chamber	Fistulae	21.25
6882	bovFT	Con_{FT}[#]	Chamber	Fistulae	22.88
6882	bovFT	FT_a	Chamber	Fistulae	20.23
6882	bovFT	FT_b	Chamber	Fistulae	20.23
7920	bovFT	Con_{FT}[#]	Chamber	Fistulae	21.51
7920	bovFT	FT	Chamber	Fistulae	20.98
1995	bovGMC	Con_G	SF_6	Stomach Pump	26.67
2028	bovGMC	Con_G	SF_6	Stomach Pump	25.37
2165	bovGMC	Con_G	SF_6	Stomach Pump	25.68
3060	bovGMC	Con_G	SF_6	Stomach Pump	24.25
4570	bovGMC	Con_G	SF_6	Stomach Pump	26.25
5679	bovGMC	Con_G	SF_6	Stomach Pump	27.32
657	bovGMC	Con_G	SF_6	Stomach Pump	25.61
6833	bovGMC	Con_G	SF_6	Stomach Pump	25.45
6857	bovGMC	Con_G	SF_6	Stomach Pump	25.96
6870	bovGMC	Con_G	SF_6	Stomach Pump	23.69
7939	bovGMC	Con_G	SF_6	Stomach Pump	27.01
1990	bovGMC	Dry_G	SF_6	Stomach Pump	21.48
2196	bovGMC	Dry_G	SF_6	Stomach Pump	19.97
2204	bovGMC	Dry_G	SF_6	Stomach Pump	18.75
2209	bovGMC	Dry_G	SF_6	Stomach Pump	19.6
3082	bovGMC	Dry_G	SF_6	Stomach Pump	20.77
4522	bovGMC	Dry_G	SF_6	Stomach Pump	20.74
5592	bovGMC	Dry_G	SF_6	Stomach Pump	20.53
5630	bovGMC	Dry_G	SF_6	Stomach Pump	19.5
5660	bovGMC	Dry_G	SF_6	Stomach Pump	18.46
6823	bovGMC	Dry_G	SF_6	Stomach Pump	21.47
1912	bovGMC	Wet_G	SF_6	Stomach Pump	24.29
2032	bovGMC	Wet_G	SF_6	Stomach Pump	22.4
2074	bovGMC	Wet_G	SF_6	Stomach Pump	21.53
2141	bovGMC	Wet_G	SF_6	Stomach Pump	19.71
3097	bovGMC	Wet_G	SF_6	Stomach Pump	19.33
4567	bovGMC	Wet_G	SF_6	Stomach Pump	19.28
6747	bovGMC	Wet_G	SF_6	Stomach Pump	20.53
6810	bovGMC	Wet_G	SF_6	Stomach Pump	21.38
6853	bovGMC	Wet_G	SF_6	Stomach Pump	21.43
7354	bovGMC	Wet_G	SF_6	Stomach Pump	21.98
1831	bovFCE	FCE	Chamber	Stomach Pump	22.87, 20.72
2287	bovFCE	FCE	Chamber	Stomach Pump	26.26, 22.56
3170	bovFCE	FCE	Chamber	Stomach Pump	21.62, 20.68
3219	bovFCE	FCE	Chamber	Stomach Pump	23.56, 25.58
441	bovFCE	FCE	Chamber	Stomach Pump	19.45, 24.98
462	bovFCE	FCE	Chamber	Stomach Pump	20.15, 22.45
463	bovFCE	FCE	Chamber	Stomach Pump	19.94, 25.81
809	bovFCE	FCE	Chamber	Stomach Pump	20.48, 23.85
818	bovFCE	FCE	Chamber	Stomach Pump	19.36, 20.68
820	bovFCE	FCE	Chamber	Stomach Pump	24.37, 23.43
822	bovFCE	FCE	Chamber	Stomach Pump	25.23, 24.42
826	bovFCE	FCE	Chamber	Stomach Pump	21.97, 22.51
841	bovFCE	FCE	Chamber	Stomach Pump	23.51, 26.27
8421	bovFCE	FCE	Chamber	Stomach Pump	21.79, 20.78
8429	bovFCE	FCE	Chamber	Stomach Pump	22.56, 24.67
8466	bovFCE	FCE	Chamber	Stomach Pump	26.18, 30.94

[†] Where multiple methane measurements are available, both are reported separated by a comma

Con_{FT} - Control diet for the bovFT experiment

FT - Treatment diet of the bovFT experiment

Con_G - Control diet for the bovGMC experiment

Dry_G - Treatment diet of the bovGMC experiment – Dry supplement

Wet_G - Treatment diet of the bovGMC experiment – Wet supplement

RF - Rumen fluid

FCE - Diet of the bovFCE animals from a lager study of feed conversion efficiency

SF_6 - A tracer gas technique for methane measurement

Chamber - A respiration chamber was used to measure methane production over 2 days

Table S2. Rumen sequence characteristics and data availability. All trimmed rumen data can be downloaded from the MG-RAST severs at ftp://ftp.metagenomics.anl.gov/projects/4126/< MG-RAST identifier >/raw . For example, the read one sequence of 2202's control diet sample can be accessed at ftp://ftp.metagenomics.anl.gov/projects/4126/4525950.3/raw . The static link to each metagenome is http://metagenomics.anl.gov/linkin.cgi?metagenome=< MG-RAST identifier > ; for example http://metagenomics.anl.gov/linkin.cgi?metagenome=4525950.3

Cow ID^	Group^	Diet^	Read Pairs*	Read 1 MG-RAST identifier	Read 2 MG-RAST identifier
2202	bovFT	$Con_{FT}^{\#}$	5 139 308	4525950.3	4525951.3
2202	bovFT	FT	6 390 148	4525948.3	4525949.3
3063	bovFT	FT	6 051 276	4525954.3	4525955.3
3063	bovFT	$Con_{FT}^{\#}$	5 938 570	4525952.3	4525953.3
6803	bovFT	FT	5 739 286	4519888.3	4519889.3
6803	bovFT	$Con_{FT}^{\#}$	5 900 689	4519873.3	4519874.3
6838	bovFT	FT	6 121 980	4519894.3	4519895.3
6852	bovFT	$Con_{FT}^{\#}$	5 829 005	4520059.3	4520060.3
6852	bovFT	FT	4 832 105	4519897.3	4519898.3
6859	bovFT	$Con_{FT}^{\#}$	5 838 894	4520065.3	4520066.3
6859	bovFT	FT	5 245 035	4520069.3	4520070.3
6882	bovFT	$Con_{FT}^{\#}$	6 076 296	4520073.3	4520074.3
6882	bovFT	FT_a	5 832 342	4520077.3	4520078.3
6882	bovFT	FT_b	4 870 694	4520079.3	4520080.3
7920	bovFT	$Con_{FT}^{\#}$	3 973 810	4520083.3	4520084.3
7920	bovFT	FT	6 044 055	4520087.3	4520088.3
1995	bovGMC	Con_G	8 570 618	4525790.3	4525791.3
2028	bovGMC	Con_G	9 530 428	4525792.3	4525793.3
2165	bovGMC	Con_G	6 994 291	4525796.3	4525797.3
3060	bovGMC	Con_G	10 199 248	4525798.3	4525956.3
4570	bovGMC	Con_G	13 438 217	4525957.3	4525799.3
5679	bovGMC	Con_G	5 385 519	4525958.3	4525800.3
657	bovGMC	Con_G	5 411 824	4525801.3	4525802.3
6833	bovGMC	Con_G	7 192 530	4525803.3	4525804.3
6857	bovGMC	Con_G	5 537 990	4525805.3	4525806.3
6870	bovGMC	Con_G	10 111 427	4525807.3	4525808.3
7939	bovGMC	Con_G	6 459 344	4525809.3	4525810.3
1990	bovGMC	Dry_G	7 069 477	4525811.3	4525812.3
2196	bovGMC	Dry_G	5 691 296	4525813.3	4525814.3
2204	bovGMC	Dry_G	6 726 286	4525815.3	4525816.3
2209	bovGMC	Dry_G	4 246 828	4525817.3	4525818.3
3082	bovGMC	Dry_G	6 083 539	4525819.3	4525820.3
4522	bovGMC	Dry_G	7 190 784	4525821.3	4525822.3
5592	bovGMC	Dry_G	4 721 060	4525823.3	4525824.3
5630	bovGMC	Dry_G	6 632 937	4525825.3	4525826.3
5660	bovGMC	Dry_G	7 101 599	4525827.3	4525828.3
6823	bovGMC	Dry_G	10 873 783	4525829.3	4525830.3
1912	bovGMC	Wet_G	6 360 807	4525831.3	4525832.3
2032	bovGMC	Wet_G	6 490 865	4525833.3	4525834.3
2074	bovGMC	Wet_G	4 539 275	4525835.3	4525836.3
2141	bovGMC	Wet_G	6 928 120	4525837.3	4525838.3
3097	bovGMC	Wet_G	4 129 699	4525839.3	4525840.3
4567	bovGMC	Wet_G	7 145 390	4525841.3	4525842.3
6747	bovGMC	Wet_G	6 978 648	4525843.3	4525844.3
6810	bovGMC	Wet_G	6 148 444	4525845.3	4525846.3
6853	bovGMC	Wet_G	4 829 180	4525847.3	4525848.3
7354	bovGMC	Wet_G	6 306 769	4525849.3	4525850.3
1831	bovFCE	FCE	8 062 738	4520089.3	4520090.3
2287	bovFCE	FCE	7 575 718	4520091.3	4520092.3
3170	bovFCE	FCE	8 605 526	4520093.3	4520094.3
3219	bovFCE	FCE	7 578 072	4520095.3	4520096.3
441	bovFCE	FCE	7 552 686	4520097.3	4520098.3
462	bovFCE	FCE	7 494 868	4520099.3	4520100.3
463	bovFCE	FCE	8 640 546	4520101.3	4520102.3
809	bovFCE	FCE	6 785 504	4520103.3	4520104.3
818	bovFCE	FCE	6 631 726	4520105.3	4520106.3
820	bovFCE	FCE	10 342 670	4520132.3	4520133.3
822	bovFCE	FCE	7 239 168	4520134.3	4520135.3
826	bovFCE	FCE	7 598 502	4520136.3	4520137.3
841	bovFCE	FCE	7 523 090	4520138.3	4520139.3
8421	bovFCE	FCE	7 100 976	4520140.3	4520141.3
8429	bovFCE	FCE	7 724 344	4520142.3	4520143.3
8466	bovFCE	FCE	7 234 222	4520144.3	4520145.3

* Number of read pairs remaining after trimming (removal of poor quality sequence)

These sequence sets are published in Ross et al 2012

^ See Table S1 for definitions

Table S3. Percentage of reads that aligned to the bovine or human metagenome database for each sample.

**Sample (Diet – CowID)**	**% aligning**	**Human sample**	**% aligning**	**Human sample**	**% aligning**
Con_G-1995	9.76	MH0001	87.50	MH0063	61.70
Con_G-2028	9.99	MH0002	78.87	MH0064	82.76
Con_G-2042	7.76	MH0003	73.84	MH0065	54.60
Con_G-2165	8.45	MH0004	58.68	MH0066	70.10
Con_G-3060	9.63	MH0005	77.19	MH0067	85.63
Con_G-4570	9.31	MH0006	72.94	MH0068	88.31
Con_G-5679	8.75	MH0007	80.85	MH0069	70.78
Con_G-657	8.68	MH0008	81.22	MH0070	70.36
Con_G-6833	8.81	MH0009	52.85	MH0071	66.64
Con_G-6857	9.24	MH0010	90.96	MH0073	84.98
Con_G-6870	9.95	MH0011	50.72	MH0074	82.09
Con_G-7939	8.62	MH0012	53.46	MH0075	78.33
Dry_G-1990	8.03	MH0013	73.01	MH0076	70.26
Dry_G -2196	8.97	MH0014	80.96	MH0077	66.56
Dry_G -2204	7.33	MH0015	82.90	MH0078	64.51
Dry_G -2209	8.65	MH0016	78.50	MH0079	45.44
Dry_G -3082	8.26	MH0017	69.41	MH0080	84.65
Dry_G -4522	9.11	MH0018	82.89	MH0081	77.76
Dry_G -5592	7.65	MH0019	88.49	MH0082	70.91
Dry_G -5630	8.54	MH0020	85.54	MH0083	78.03
Dry_G -5660	8.09	MH0021	76.58	MH0084	82.80
Dry_G -6823	7.18	MH0022	53.76	MH0085	86.55
Wet_G-1912	7.28	MH0023	78.09	MH0086	75.69
Wet_G -2032	7.92	MH0024	70.55	O2.UC.11	75.61
Wet_G -2074	7.35	MH0025	63.40	O2.UC.12	61.35
Wet_G -2141	7.71	MH0026	78.88	O2.UC.13	69.72
Wet_G -3097	8.59	MH0027	84.25	O2.UC.14	75.33
Wet_G -4567	8.43	MH0028	80.13	O2.UC.16	59.66
Wet_G -6747	7.87	MH0030	59.88	O2.UC.17	83.25
Wet_G -6810	8.30	MH0031	48.02	O2.UC.18	81.76
Wet_G -6853	7.73	MH0032	79.11	O2.UC.19	76.85
Wet_G -7354	6.26	MH0033	78.31	O2.UC.1	71.78
FCE -1831	4.71	MH0034	83.49	O2.UC.20	82.74
FCE -2287	2.90	MH0035	70.11	O2.UC.21	91.79
FCE -3170	1.94	MH0036	71.41	O2.UC.22	85.74
FCE -3219	3.46	MH0037	74.11	O2.UC.23	74.80
FCE -441	4.16	MH0038	73.73	O2.UC.24	64.56
FCE -462	6.50	MH0039	85.36	O2.UC.4	76.59
FCE -463	4.93	MH0040	67.34	V1.CD.12	89.02
FCE -809	5.42	MH0041	82.96	V1.CD.13	71.61
FCE -818	5.05	MH0042	53.74	V1.CD.14	80.45
FCE -820	6.27	MH0043	77.60	V1.CD.15	87.28
FCE -822	4.61	MH0044	79.26	V1.CD.1	92.44
FCE -826	8.29	MH0045	80.05	V1.CD.2	75.45
FCE -841	3.40	MH0046	60.51	V1.CD.3	58.44
FCE -8421	5.95	MH0047	34.79	V1.CD.4	82.21
FCE -8429	4.12	MH0048	76.29	V1.CD.6	88.05
FT -2202	6.77	MH0049	79.44	V1.CD.8	74.99
Con_{FT} -2202	6.06	MH0050	65.35	V1.CD.9	77.36
FT -3063	6.78	MH0051	72.99	V1.UC.10	73.76
Con_{FT} -3063	5.15	MH0052	54.10	V1.UC.13	69.87
FT -6803	5.02	MH0053	50.82	V1.UC.14	67.62
Con_{FT} -6803	5.63	MH0054	67.12	V1.UC.15	76.03
FT -6838	6.91	MH0055	57.88	V1.UC.17	67.89
FT -6852	5.98	MH0056	70.94	V1.UC.18	78.70
Con_{FT} -6852	7.79	MH0057	51.62	V1.UC.19	77.98
FT -6859	7.08	MH0058	80.95	V1.UC.21	80.31
Con_{FT} -6859	5.63	MH0059	74.96	V1.UC.6	79.05
FT -6882_a	3.91	MH0060	51.62	V1.UC.7	85.26
FT -6882_b	5.11	MH0061	86.70	V1.UC.8	80.85
Con_{FT} -6882	4.53	MH0062	76.98	V1.UC.9	78.94
FT -7920	5.58				
Con_{FT} -7920	8.58				

Con_{FT} - Control diet for the bovFT experiment
FT - Treatment diet of the bovFT experiment
Con_G - Control diet for the bovGMC experiment
Dry_G - Treatment diet of the bovGMC experiment – Dry supplement
Wet_G - Treatment diet of the bovGMC experiment – Wet supplement
FCE - Diet of the bovFCE animals from a lager study of feed conversion efficiency

a) An example metagenomic profile matrix

```
Grapemarc-Con-3060 33 49 4 14 4 37 2 28 31 78 28 8 24 321
Grapemarc-Con-5679 37 80 3 1 4 46 2 46 41 140 55 6 38 176
Grapemarc-Con-6870 50 49 3 4 0 41 2 48 46 91 284 2 20 77
Grapemarc-Dry-1990 49 59 4 2 2 53 6 50 22 74 44 4 28 247
Grapemarc-Dry-3082 32 50 2 5 10 56 11 38 11 76 74 4 4 230
Grapemarc-Dry-5592 52 79 3 3 6 94 0 76 46 132 84 4 32 372
Grapemarc-Wet-1912 29 68 0 4 4 58 0 48 34 109 34 0 14 272
Grapemarc-Wet-2074 40 49 4 4 6 81 2 83 26 85 62 4 36 515
Grapemarc-Wet-6747 26 68 7 2 7 57 10 49 44 94 40 4 15 130
Grapemarc-Wet-6853 24 44 2 6 8 63 0 59 25 128 76 4 20 190
6803-control-rumen 35 57 0 0 0 160 2 95 37 224 18 0 31 146
6859-control-rumen 42 69 2 2 2 114 4 83 30 217 70 0 50 192
3063-FT-rumen 19 42 0 4 0 121 0 108 38 197 109 0 9 89
6838-FT-rumen 37 77 0 2 0 229 0 241 38 207 4 0 36 108
7920-FT-rumen 35 31 0 0 2 89 4 74 24 209 79 2 28 113
FCE-2287 4 22 6 10 16 8 12 8 8 87 56 6 4 8
FCE-462 4 21 34 40 25 9 52 8 12 55 56 45 4 30
FCE-809 9 38 44 51 58 16 56 20 16 93 30 39 8 34
FCE-826 6 8 54 65 90 10 67 0 8 32 12 51 4 40
FCE-8421 6 22 40 63 75 17 44 18 0 67 24 56 8 29
```

b) An example phenotype file

```
Grapemarc-Con-3060 0.6403346
Grapemarc-Con-5679 1.741141
Grapemarc-Con-6870 0.439536
Grapemarc-Dry-1990 -0.3529012
Grapemarc-Dry-3082 -0.6074851
Grapemarc-Dry-5592 -0.6935416
Grapemarc-Wet-1912 0.6546774
Grapemarc-Wet-2074 -0.3349727
Grapemarc-Wet-6747 -0.6935416
Grapemarc-Wet-6853 -0.3708296
```

c) An example output file from rrBLUP based predictions

```
6803-control-rumen 3.62880808782934e-09
6859-control-rumen 4.39322176464028e-10
3063-FT-rumen 3.03241348974004e-09
6838-FT-rumen 3.22750950791945e-09
7920-FT-rumen 1.07344093845841e-09
FCE-2287 -1.70532194468503e-09
FCE-462 -2.84758459821551e-09
FCE-809 -3.09477993937246e-09
FCE-826 -3.00581497279512e-09
FCE-8421 -3.82382100268925e-09
```

d) An example .as file generated by the ASReml script

```
MetagenomicPredictions
 name !ALPHA 10 !LL 19
 phenotype
 id 10
MPM.txt.giv
Phenotypes.txt.phen !AISING
phenotype ~ mu !r giv(id,1)
```

Figure S1. Examples of metagenomic prediction files. Both input files are space delimited and the names in the phenotype file match the names in the profile matrix exactly. a) metagenome profile matrix which contains counts of the number of reads which align to each contig from each sample, b) the phenotypes of the reference population which will be used to predict the unknown samples. All samples in the phenotype file should be in the metagenome profile matrix; but not all samples in the metagenome profile matrix need be in the phenotype file (i.e. the validation animals are left out of the phenotype file) c) example of phenotypes predicted by the rrBLUP method, the file which these values are written to is determined by the third variable given to the script, d) example of the .as file that is generated from the ASReml method. This file and the others generated is then used to run ASReml.

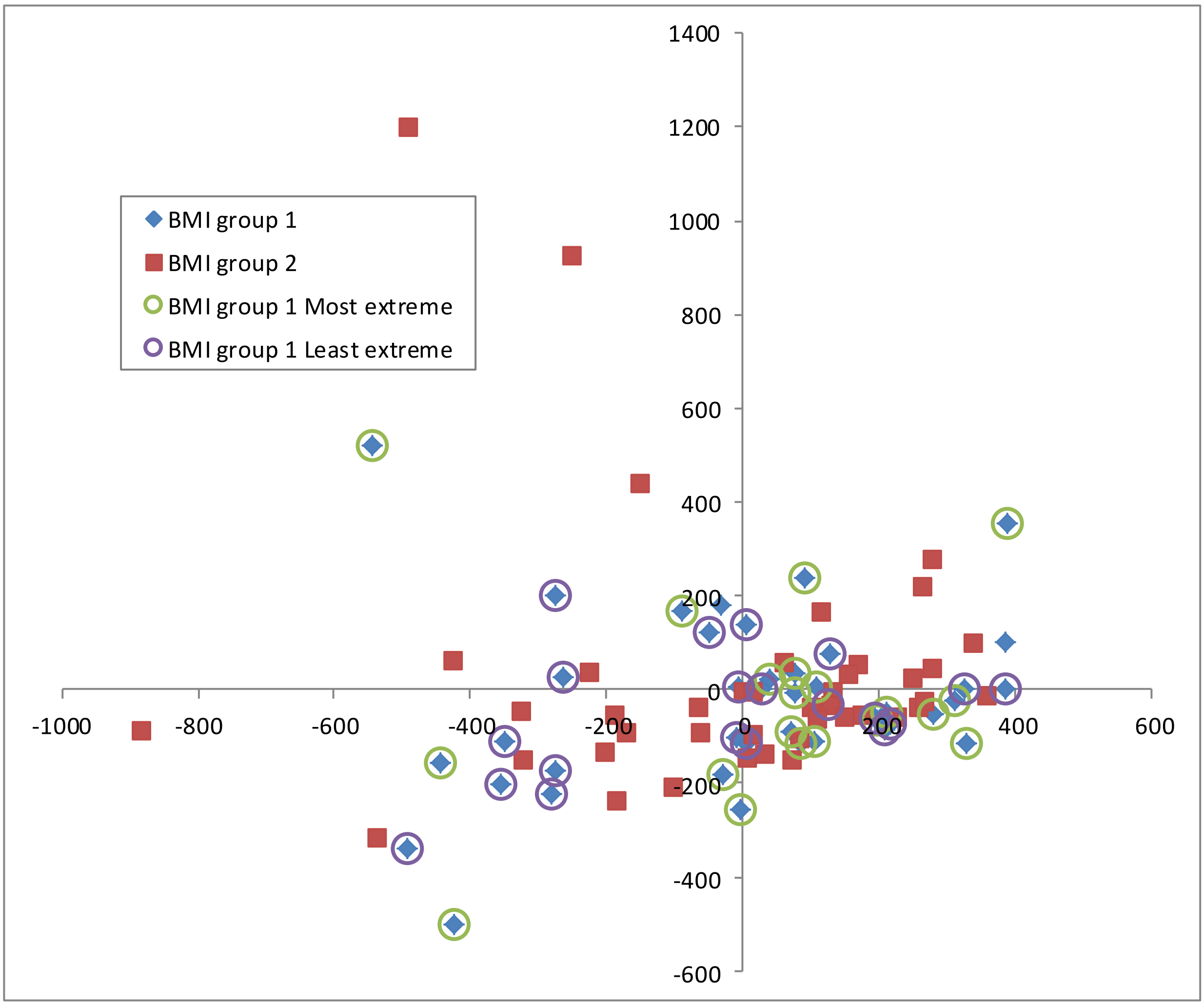

Figure S2. A principle componant plot of the human BMI datasets. Principle componet 1 is shown on the x axis, principle component 2 is shown on the y axis. Different subsets used for validation are indicated by different symbols. The most and least extreme phenotypes of the BMI group 1 dataset are circled.

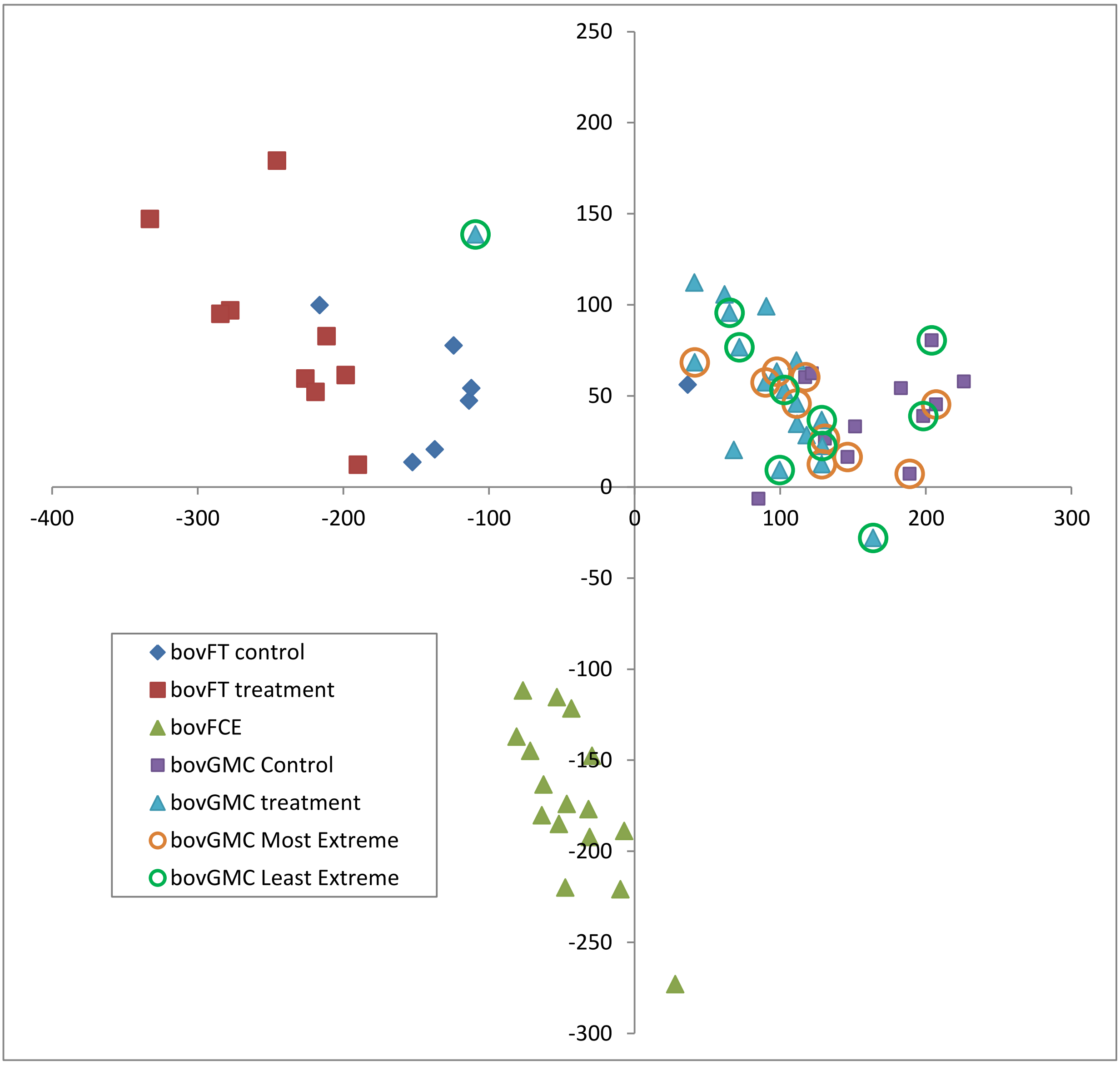

Figure S3. A principle componant plot of the bovine rumen datasets. Principle componet 1 is shown on the x axis, principle component 2 is shown on the y axis. Different subsets used for validation are indicated by different symbols. The most and least extreme phenotypes of the bovGMC dataset are circled.

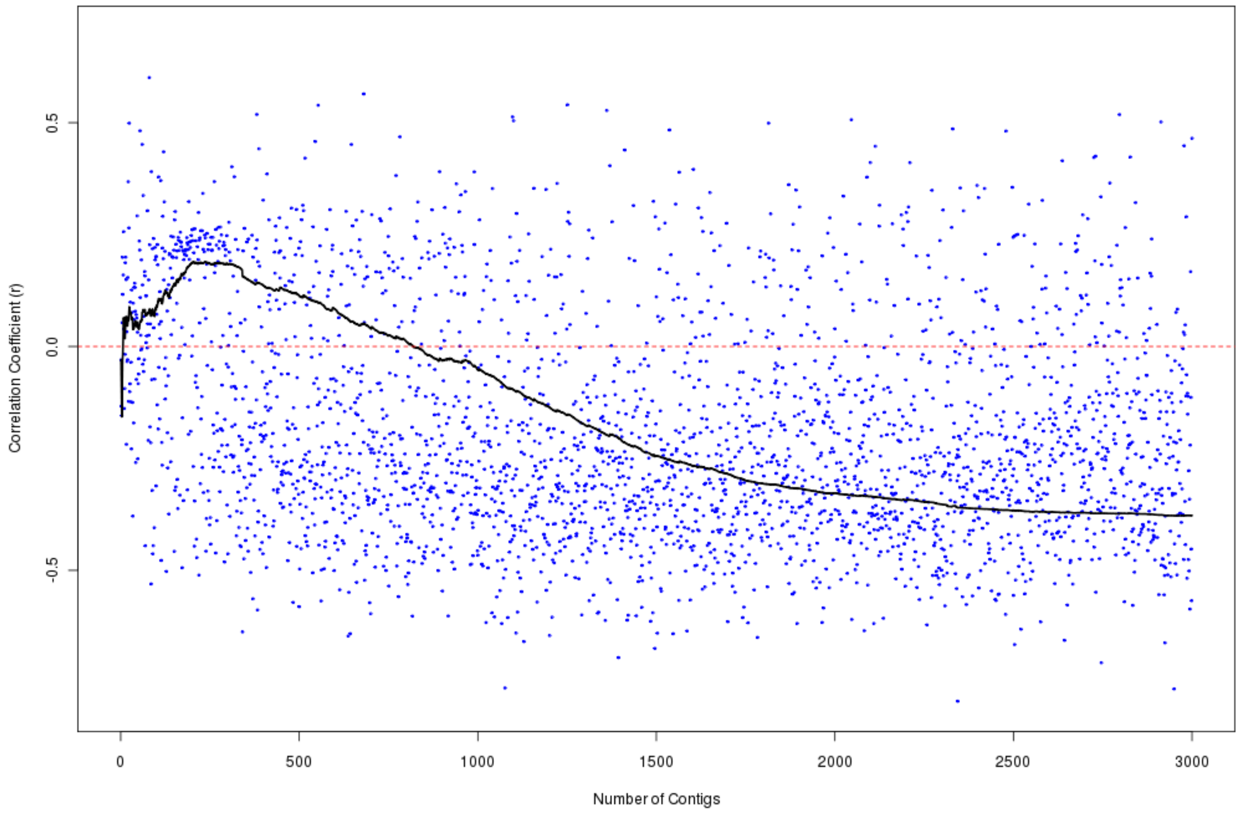

Figure S4. Prediction by contig. Correlations between predicted and measured methane production. The X axis is contigs ordered from most significant to least significant from a linear model (methane ~ contig abundance), with metagenomic profiles from both bovFT and bovGMC used in the model. The resulting equation was then applied to the bovFCE dataset to predict methane production. The Blue dots indicate the correlation coefficient between predicted methane using that one contig, and actual methane. The black line is the sum of the predictions from the x most significant contigs. Predictions have an average negative correlation with real methane production. This may be an artefact of the methane mitigating diet effects.

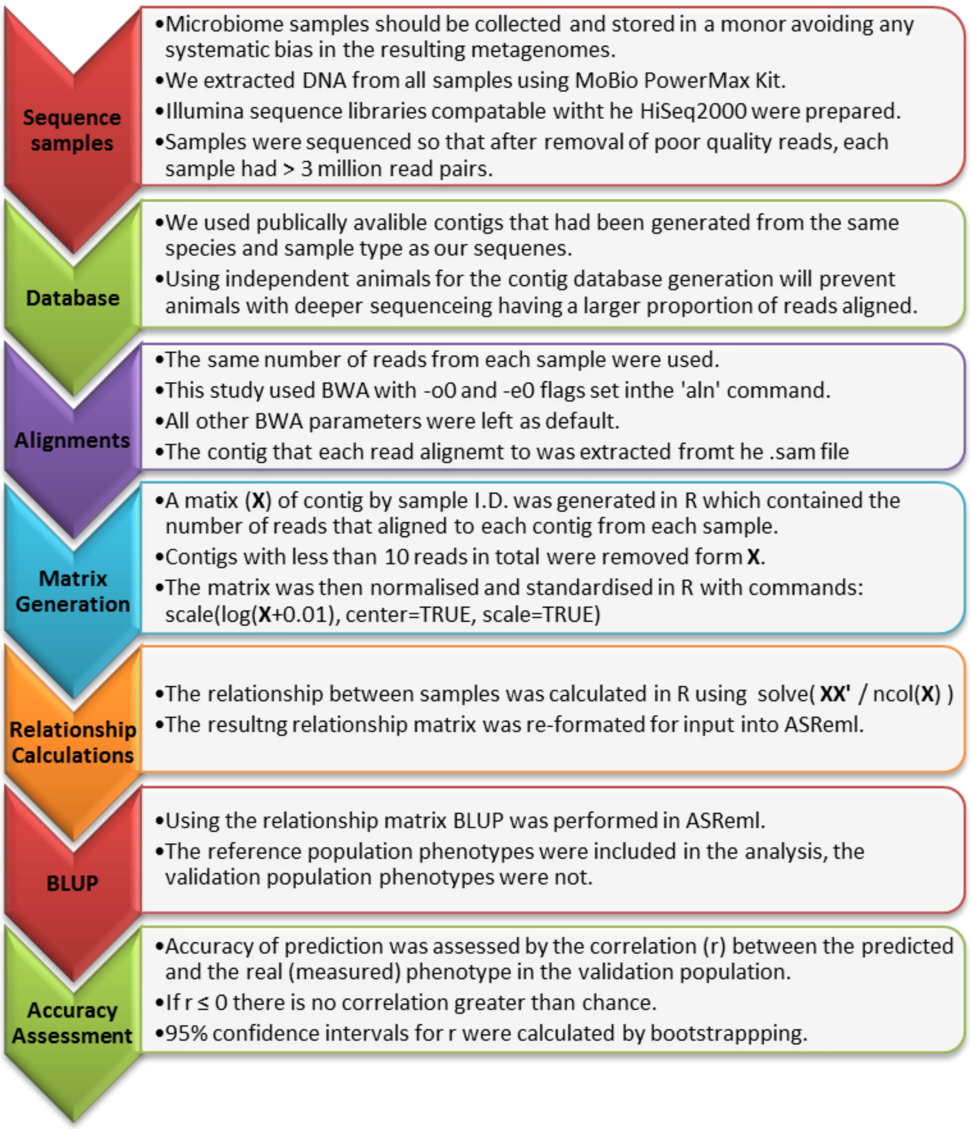

Figure S5. Basic methodological procedure of performing metagenomic predictions. R refers to the R statistical language [3]. BWA is a short read alignment program [9].

References

1. Gilmour AR, Gogel BJ, Cullis BR, Thompson R (2006) ASReml User Guide Release 2.0. VSN International Ltd, Hemel Hempstead, HP1 1ES, UK.
2. Endelman JB (2011) Ridge regression and other kernels for genomic selection with R package rrBLUP. The Plant Genome 4: 250-255.
3. R Development Core Team (2011) R: A Language and Environment for Statistical Computing. Vienna, Austria: R Foundation for Statistical Computing.
4. Tan ND, Wanapat M, Uriyapongson S, Cherdthong A, Pilajun R (2012) Enhancing Mulberry Leaf Meal with Urea by Pelleting to Improve Rumen Fermentation in Cattle. Asian Australasian Journal of Animal Science 25: 452-461
5. Park K, Jang J, Irimia D, Sturgis J, Lee J, et al. (2008) 'Living cantilever arrays' for characterization of mass of single live cells in fluids. Lab Chip 8: 1034-1041.
6. Janloo S, Dolezal H, Strasia C, Clarkson W, Owens F, et al. (1998) Impact of withholding feed on weight and composition of ruminal contents. Stillwater, OK: Oklahoma State University. Avalible at http://www.ansi.okstate.edu/research p.
7. Craig WM, Broderick GA, Ricker DB (1987) Quantitation of Microorganisms Associated with the Particulate Phase of Ruminal Ingesta. The Journal of Nutrition 117: 56-62.
8. Savage DC (1977) Microbial ecology of the gastrointestinal tract. Annual Review of Microbiology 31: 107-133.
9. Li H, Durbin R (2009) Fast and accurate short read alignment with Burrows–Wheeler transform. Bioinformatics 25: 1754-1760.
